# Pediatricians' perspectives on the impact of MRSA in primary care: a qualitative study

**DOI:** 10.1186/1471-2431-9-27

**Published:** 2009-04-14

**Authors:** Adam L Hersh, Michael D Cabana, Ralph Gonzales, Budd N Shenkin, Christine S Cho

**Affiliations:** 1 Division of Pediatric Infectious Diseases, University of California, San Francisco, San Francisco, CA, USA; 2 Division of General Pediatrics, University of California, San Francisco, San Francisco, CA, USA; 3 Division of General Internal Medicine, University of California, San Francisco, San Francisco, USA; 4 Division of Emergency Medicine, Children's Hospital and Research Center Oakland, Oakland, CA, USA; 5 President, Bayside Medical Group, Oakland, CA, USA

## Abstract

**Background:**

The incidence of skin and soft-tissue infections (SSTIs) has rapidly increased among children in primary care settings since the emergence of community-associated methicillin-resistant *Staphylococcus aureus *(CA-MRSA). Recent treatment recommendations emphasize CA-MRSA as the primary cause, performing incision and drainage (I&D) as the primary therapy, and not prescribing antibiotics for uncomplicated cases. It is unknown how this epidemic has impacted primary care pediatricians in terms of their practice patterns and barriers they face to providing recommended therapies.

**Methods:**

3 Focus groups among 29 primary care pediatricians in the San Francisco Bay Area were conducted. Transcripts were reviewed and coded into major themes by two investigators using modified grounded theory.

**Results:**

Substantial changes in clinical practice have occurred since the emergence of CA-MRSA. These include increased office visits for SSTIs, patients with multiple recurrences and transmission within households. Additionally, our participants reported increased visits for mild skin problems due to media reports contributing to fears about CA-MRSA. Participants routinely prescribed antibiotics for SSTIs, however, few performed I&D. Few were aware of recent SSTI treatment recommendations. Barriers to prescribing antibiotics with CA-MRSA activity included concerns about side-effects and lack of local epidemiologic data showing that it is the primary etiology. Barriers to performing I&D included lack of training, resources and skepticism about its necessity. Important clinical challenges included increased time demands for follow-up visits and patient education along with the lack of evidence-based strategies for preventing recurrent inections and household transmission.

**Conclusion:**

CA-MRSA has influenced the presentation and treatment of SSTIs especially in terms of case numbers and recurrences. Barriers to providing recommended therapies can be addressed through improved dissemination of treatment guidelines and epidemiologic data. Studies are urgently needed toimprove theevidence-base for treatment and prevention strategies.

## Background

Community-associated methicillin-resistant *Staphylococcus aureus *(CA-MRSA) first emerged among children in the 1990s [1]. Over the last decade, CA-MRSA has become the predominant cause of purulent skin and soft-tissue infections (SSTIs) in the United States [2]. Although invasive infections occur, SSTIs are the most common clinical manifestation of CA-MRSA [3]. Coincident with this epidemic, the incidence of pediatric ambulatory visits for SSTIs has nearly tripled, evidence that CA-MRSA has significantly increased the health care burden of SSTIs [4]. Following a 2007 publication about the epidemiology of invasive MRSA infections [5] and highly publicized deaths of healthy adolescents, media attention to MRSA has increased substantially [6].

Therapeutic options for SSTIs include antibiotic therapy and incision and drainage (I&D). Recommendations from both the Centers for Disease Control (CDC) [7,8] and the American Academy of Pediatrics (AAP) [9] emphasize performing I&D as the primary treatment, sending purulent material for culture, not using antibiotics for uncomplicated cases and targeting CA-MRSA when empiric antibiotics are prescribed. These guidelines assume that primary care physicians have a system to monitor community epidemiology, track culture results and have sufficient resources to perform I&D.

We hypothesized that the increased incidence, complexity and public attention to CA-MRSA have placed a significant burden on primary care pediatricians' practices. We conducted focus groups with primary care pediatricians to assess the impact of CA-MRSA on SSTI management and to identify barriers to providing recommended therapies.

## Methods

### Study Design

Because the CA-MRSA epidemic is recent and the impact on pediatricians is largely unknown, we used a qualitative focus group design to obtain in-depth information directly from the key stakeholders about their experience and practice patterns for patients with purulent SSTIs. We conducted three focus groups of primary care pediatric practitioners in the San Francisco Bay Area to understand the impact of CA-MRSA on the presentation and management of purulent SSTIs. San Francisco is a city with 110,000 persons under age 18 and has a high prevalence of CA-MRSA. A recent study incorporating laboratory data from 8 of the 9 medical center laboratories in San Francisco estimated the annual CA-MRSA incidence as 316 cases per 100,000 population [10]. In San Francisco, CA-MRSA is the predominant organism among cultured abscesses (80% at the University of California, San Francisco pediatric clinic) [11,12] which mirrors national trends [2]. The study was approved by the Committee on Human Research at the University of California, San Francisco and the Institutional Review Board at the Children's Hospital and Research Center Oakland.

### Participant Recruitment

We recruited participants by direct mailings utilizing a database of all general pediatricians in San Francisco (n = 113) maintained by the University of California, San Francisco Division of General Pediatrics. This database is continuously updated with names and contact information of all general pediatricians in San Francisco. Additionally, we sent direct mailings to 2 large East Bay Area practices. Participants were eligible if they were primary care, office-based pediatric practitioners in full-time clinical practice. According to standard qualitative design, purposive sampling (deliberate selection of participants to maximize demographic variability and minimize response bias) was used to select participants with diverse experiences and opinions. Participants were continuously recruited and focus groups were conducted until content saturation was achieved. Participants were provided dinner, an honorarium of $100 and educational materials about CA-MRSA after completion of the focus group. Funding for the focus groups was provided by the University of California, San Francisco, Division of General Pediatrics.

### Conducting the Focus Groups

**Figure 1:**
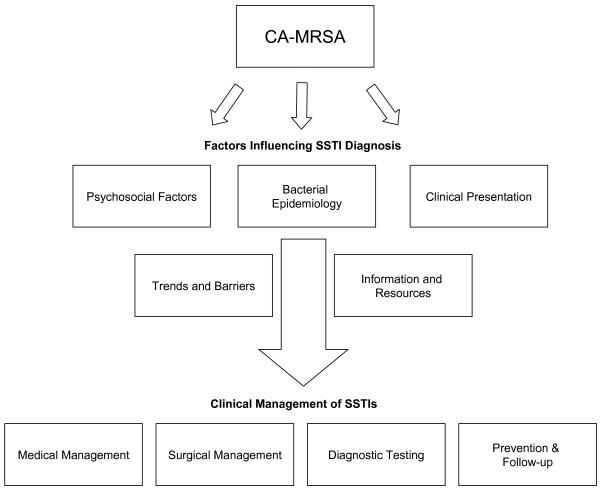
**Conceptual model of the impact of community-associated methicillin-resistant *Staphylococcus aureus *(CA-MRSA) on primary care**. The top level reflects how CA-MRSA influences factors relating to diagnosis and presentation of SSTIs. The second level accounts for how trends in the epidemiology of SSTIs, barriers to implementing recommended therapies and information/resources available to physicians influence the clinical management of SSTIs. SSTI, skin and soft-tissue infection.

In order to allow participants to share their viewpoints in an unbiased manner, we developed a guide of open-ended questions to assess the impact of CA-MRSA at the community pediatrician level. The question guide was based on a conceptual model developed specifically for this study (Figure 1). The conceptual model was derived from insight from a prior study about how patients with SSTIs are managed in office settings [4], a pilot focus group and our own clinical experiences. The guide addressed three general topics (1) the clinical presentation and management of SSTIs and how this may have changed since the emergence of CA-MRSA; (2) knowledge and awareness of AAP/CDC treatment recommendations and how they keep up to date about SSTIs; and (3) barriers to providing recommended therapies. It was revised after independent review by three investigators (ALH, MDC, CSC), and testing with a pilot focus group.

Three semi-structured focus groups lasting one-hour based on the question guide were moderated by one investigator (CSC). The focus groups were recorded using non-concealed microphones, participants were informed that the transcripts would not contain personal identifiers and they could refuse to answer any questions. A second investigator (ALH) attended all focus groups to ensure that all topics were covered and took notes on general themes and participant reactions and interactions. Participants were encouraged to express all viewpoints and to respond to other participants' comments.

### Analysis

Audiotapes were transcribed verbatim except for removal of identifying information. Two investigators (ALH, CSC) independently reviewed the transcripts to minimize bias. Initially, both investigators (who attended all focus groups) discussed impressions, reviewed notes, and read all transcripts to create a coding template of major themes that arose during all three focus groups. Transcripts were then reread by both investigators and using constant comparative techniques based on grounded theory, participant responses were independently coded into thematic categories. Coding by each investigator was then compared and discordant classifications were discussed and resolved.

## Results

### Participants

Three focus groups of 8–11 participants were conducted during January/February 2008. Participants included 28 pediatricians and 1 pediatric nurse practitioner (Table 1). The major themes identified from the focus groups are summarized in Table 2 and discussed below.

**Table 1:** Demographic and practice characteristics of study participants (total N = 29).

Characteristic	Number (%)
Male	9 (31)
Mean age in years (range)	44 (29–72)
Mean years practice (range)	14 (2–43)
Practice Setting	
Private practice	20 (69)
University practice	3 (10)
Health maintenance organization	3 (10)
Community health clinic	3 (10)

**Table 2:** Summary of major themes identified about the impact of CA-MRSA on primary care pediatric practices for SSTIs.

	Themes
How has your practice changed since the emergence of CA-MRSA?	
Bacterial Epidemiology	Majority is CA-MRSA.
Clinical Presentation	Increased case numbers but not severity. Increased recurrences, household transmission and incidence among infants. Substantial patient fear of CA-MRSA.
Clinical Management	
Medical Management	Antibiotics are highly important and routinely prescribed. Some increased empiric prescribing of antibiotics that treat CA-MRSA.
Surgical Management	Variable perception of importance of I&D.
Diagnostic Testing	Increased use of bacterial culture.
Prevention and Follow-up	More aggressive follow-up. Uncertainty about role of decolonization.
What is your knowledge about SSTI treatment recommendations and how do you stay updated?	
	Limited awareness of published recommendations from CDC and AAP.
What are the barriers to compliance with current recommendations?	
	Internal Barriers
	Lack of awareness of current guidelines.
	Inexperience with antibiotics that treat CA-MRSA.
	Lack of self-efficacy with I&D.
	Skepticism about treatment with I&D alone.
	External Barriers
	Concerns about antibiotic compliance.
	Lack of epidemiologic data about resistance patterns for SSTIs.
	Lack of resources to perform office I&D.
	Delays in receiving culture results.

### Changes in practice since emergence of CA-MRSA

Six themes emerged related to changes in clinical practice.

#### Bacterial Epidemiology

Most participants identified CA-MRSA as the most common cause of purulent SSTIs in their practices.

"I feel like they [cultures] all come back MRSA."

#### Clinical Presentation

Most participants felt that the incidence of purulent SSTIs had recently increased due to CA-MRSA. However, there was consensus that the severity of SSTIs had not changed and that patients with CA-MRSA typically respond to treatment. Two clinical features were highlighted as important changes associated with CA-MRSA. The first was patients with multiple recurrences and secondary transmission to their household members.

"Just recurrence, recurrence, recurrence, where it's the same kid, the same family, over and over again."

The second was increased SSTI incidence among infants, especially in the form of pustulosis.

Nearly all participants reported that patient and family fears about CA-MRSA is a major sentiment in their practices. Participants believed that this has been magnified by recent media reports describing CA-MRSA as a "super-bug."

"Staph has been around for forever, but now it has a totally new connotation. They automatically think it's life threatening. I hear, "They have the 'Staph?' The Staph that's deadly?" And [since] those major stories that have hit – everybody thinks coming in with a little abscess ... it's going to be fatal."

Physicians reported that parent concerns were reinforced by procedures required by patients' schools.

"Now I have to write notes saying that this kid can go back to school with their staph infection."

Many reported that patient and family fears about CA-MRSA were responsible for office visits for mild skin problems that would not have otherwise prompted medical attention. Most felt that media reports were inaccurate and exaggerated.

"It comes up all the time. People are always asking about it. Every time there's some little scratch, they bring them in because they're worried it's Staph."

#### Medical Management

All participants routinely prescribe oral antibiotics for purulent SSTIs and stated that they prescribe antibiotics independent of performing I&D. This practice preceded the emergence of CA-MRSA.

"I always put them on an oral antibiotic. I don't leave it up to God to do it."

Some reported that now they empirically prescribe antibiotics with activity against CA-MRSA (e.g. trimethoprim/sulfamethoxazole or clindamycin) for patients with purulent SSTIs whereas they previously used cephalexin, while other participants still chose cephalexin as a first-line antibiotic. Participants were not aware of community rates of resistance to these antibiotics and thus resistance data was not an important factor in their antibiotic selection. For cases of pustulosis in infants, some participants reported prescribing only topical therapy with mupirocin.

#### Surgical Management

Owing to the increased incidence of purulent SSTIs in their practices, some participants reported that they now perform I&D more frequently, however, overall use of I&D is low. There was variation in the perception of the importance of I&D. Some participants questioned whether this procedure was necessary for all patients, especially if they had been treated with appropriate antibiotics.

"I believe a lot of these can be treated without I&Ding ... you're giving antibiotics."

"I'll start them on something (antibiotics) and then follow-up. I find that I'm able to sort of manipulate around it because it's not that urgent."

Although a few participants perform I&D of deeper lesions and use packing, most only perform very limited procedures (e.g. a small incision or nick with a needle or scalpel).

#### Diagnostic Testing

Most participants reported performing cultures of purulent SSTIs routinely which is a change in practice due to CA-MRSA.

"10 years ago I wouldn't culture on a routine basis ... I culture everything now."

Participants mentioned that there are circumstances where cultures are not routinely performed, such as recurrent infections or lesions that appear to be resolving. No participants reported performing cultures for the benefit of epidemiologic surveillance at the practice or community level.

#### Prevention and Follow-up

Many participants expressed uncertainty about the benefits of prevention measures, especially decolonization regimens and reported frustration with the apparent lack of consensus about their value.

"I feel like I'm going around in a circle, treat the family, don't treat the family. I don't know what to do anymore."

Nonetheless, many participants reported prescribing mupirocin for nasal decolonization for index patients and their family members, especially in cases of recurrences.

Many participants noted that managing patients with CA-MRSA is extremely time consuming. They noted spending significant time addressing parental fears, answering questions and explaining decolonization regimens. Although only a few mentioned emphasizing personal and environmental hygiene, those that did indicated that it also was very time-consuming. Many participants mentioned that due to CA-MRSA, they now routinely arrange for their patients to follow-up within 24 hours after treatment initiation. This is a recent change in practice.

"In the past I would never follow-up a regular abscess. Now I always have them come back, no matter what."

### Knowledge about current SSTI treatment recommendations and how to stay updated

There is very limited awareness of recently published SSTI treatment recommendations. Only 1 of 29 participants was aware of the CDC recommendations and 1 was aware of the AAP recommendations.

For resources about local epidemiology, most participants were aware of hospital antibiograms (cumulative reports of antibiotic susceptibility for bacteria). However, there was consensus that this information is not helpful for treating their patients with SSTIs because they do not believe it is a valid reflection of their outpatient population. There was strong desire to receive more clinically relevant antibiograms based on outpatient cultures and sorted by specimen type (e.g. abscess, urine etc).

Participants mentioned many resources they use to stay updated about treatment recommendations (Table 3). Most participants rely on electronic sources including journals and websites. Several indicated that they value input from infectious disease specialists but have limited access to them.

**Table 3:** Important sources of information and resources about management of skin and soft-tissue infections.

Electronic sources
Peer reviewed and non-peer reviewed journals
Peer reviewed websites (e.g. UpToDate)
MD Consult
SERMO
Non-peer reviewed print journals (e.g. Infectious Diseases in Children)
Television and print media
Infectious Disease consultants
Health Department bulletins
Grand Rounds, Continuing Medical Education

### Barriers to compliance with current recommendations

Participants mentioned multiple barriers that make adherence to treatment recommendations challenging. These included both internal barriers (related to personal levels of knowledge, attitude and skills) and external barriers (related to the healthcare system) [13].

#### Medical management

In terms of recommendations to target CA-MRSA with antibiotic therapy, participants mentioned a variety of external barriers limiting willingness to adhere to this recommendation. These include concerns about compliance with antibiotic therapy (related to taste and dosing frequency), cost and insurance coverage, desire for Group A *Streptococcus *coverage, promoting further resistance and causing adverse drug events (e.g. *Clostridium difficile *colitis and Stevens-Johnson Syndrome). Additionally, some felt that their limited experience using trimethoprim/sulfamethoxazole and clindamycin contributed to reluctance to prescribe these medications. The lack of readily available data about the epidemiology of SSTIs, including rates of resistance to specific antibiotics (e.g. clindamycin) in the community is also felt to be an important barrier to adopting this recommendation.

#### Surgical management

Several barriers to performing office I&D were shared by participants. Many of these are external and relate to system-based challenges inherent to performing a procedure in a busy office practice. These include inadequate time in the schedule and insufficient resources or personnel to provide pain control and sedation, gather supplies and to hold the patient.

"It (I&D) puts you behind."

Several participants mentioned a lack of training and limited expertise in performing I&D as an important internal barrier.

"Why not put it in the hands of somebody who does a lot of I&Ds."

"I don't feel like I'm good at it."

Further, some participants are uncomfortable performing invasive procedures in the office because it conflicts with parent expectations of an outpatient office setting (vs. another setting such as an emergency department).

"...you're trying to I&D a 2 year old who's screaming. That spreads out to the other patients and it's an inappropriate thing to do."

"I think it's tough for the relationship with the kid doing those things (I&D)."

On the other hand, a few participants felt strongly about performing the procedure themselves because they believed that other physicians may not appreciate the unique needs of children.

In terms of the recommendation to treat some purulent SSTIs with I&D alone, several participants were skeptical about applying this strategy in routine practice.

"You pick up Time Magazine and they're saying more people are going to die of MRSA than HIV in the next 10 years. It makes it hard to feel comfortable just draining it."

#### Diagnostic testing

Participants mentioned that there are barriers related to performing and using culture results. In some cases, participants are concerned about the additional cost to their patients while others mentioned that delays exceeding 2–3 days in receiving culture results are common. Others reported that not all laboratories perform D-testing for inducible clindamycin resistance. Most participants noted that the follow-up for cultures is labor-intensive.

"It's more work – culturing and calling and following-up and making sure they're on the right medicine."

## Discussion

We found that the emergence of CA-MRSA has influenced multiple factors related to the diagnosis and clinical management of SSTIs in ambulatory pediatrics. Additionally, we found that there were both internal and external barriers [13] to adoption of recommended therapies for SSTIs and opportunities to improve care. Our findings highlight the complex issues facing physicians when a new disease emerges that generates public attention, affects a large number of patients and requires changes in practice.

This is the first study to explore the significance of CA-MRSA in primary care pediatrics in terms of the impact it has had on clinical practice. The qualitative design of our study enabled us to probe with great depth around issues faced by primary care physicians and their practice patterns. Similar to national trends, our participants reported increased patient visits due to SSTIs caused by CA-MRSA [4]. As a consequence, some of the challenges identified related to increased time demands in terms of changes in how pediatricians manage children with SSTIs in the CA-MRSA era. This includes time for more follow-up visits, following up on culture results and time requirements for explaining decolonization regimens to families. Additionally, non-medical burdens were noted such as frustration about the lack of evidence-based strategies for preventing recurrences and household transmission as well as time demands to address questions and concerns about CA-MRSA among patients and their families.

Addressing these challenges faced by primary care pediatricians is crucial to informing the design of future interventions focused on treatment and prevention of CA-MRSA.

Participants in our study believe that fear induced by media coverage of CA-MRSA caused increased visits for mild skin problems. We did not directly ascertain from our participants whether they felt pressure to prescribe antibiotics for mild infections, however, participants reported using antibiotics frequently and substantially increasing the number of cultures they performed. The potential for media to influence physician behavior was highlighted in a prior study showing a strong association between media reports of invasive group A streptococcus infection and testing for GAS in a pediatric ED [14].

The potential for "disease mongering" (expansion of the market for a disease) exists because of the broad spectrum of severity for CA-MRSA ranging from life-threatening (invasive infections) to mild (most SSTIs) to none (asymptomatic colonization). Media reports prompted by severe infections may "blur the distinction between mild and severe," which is a key component of this phenomenon [15]. Determining whether the observations noted by our participants are representative of national trends will further our understanding of how the media can influence patient use of ambulatory services and subsequent physician behavior. Gaining more direct insight into how communication from schools to parents about CA-MRSA influences office visits is important since our participants noted that schools also contributed to parental fears and concerns. Additionally, the impact of new legislation mandating MRSA screening for hospital admissions in many states (including California) on public perceptions about MRSA remains unknown.

Our participants felt strongly about prescribing antibiotics for SSTIs, independent of whether or not I&D is performed. Although current recommendations suggest that some patients can be treated with I&D alone, our results indicate reluctance among primary care pediatricians to adopt this practice. Some of the reluctance to limit antibiotic use may stem from the challenges faced by primary care pediatricians to performing adequate I&D in office settings including internal barriers such as a lack of self-efficacy and guideline agreement. Additionally, external barriers such as inadequate time and supplies and inability to provide adequate pain control or sedation also seem to influence their practice. Our participants did not explicitly cite reimbursement as a barrier to performing I&D, however, reimbursement has been previously shown to be a barrier to guideline adherence [16]. Participants were concerned about the time and supplies that are required, issues that could impact reimbursement by increasing overhead or decreasing patient flow in a busy office practice. Designing interventions to increase use of I&Ds for purulent SSTIs is important because for some patients, failure to perform a prompt I&D may delay resolution of the infection.

Some participants in our study did not target CA-MRSA with their initial empiric antibiotic. Although the optimal antibiotic therapy for purulent SSTIs is unknown, experts have suggested using a 10% community prevalence as a threshold above which to empirically treat with an antibiotic active against CA-MRSA and this was validated using a decision-analytic model [11,17]. Our prior work [4] has shown that cephalexin remains commonly prescribed for SSTIs despite the fact that CA-MRSA prevalence in most communities is >10%. Participants in our study felt that they did not have access to epidemiologic data about bacterial infections and antibiotic resistance patterns in their community. Because physician education has been shown to improve antibiotic selection [18], it is possible that direct dissemination of epidemiologic data about SSTIs to pediatricians would address this barrier and could influence their prescribing.

Most of the participants in our study were unaware of recent SSTI treatment recommendations. Since guidelines are more credible to physicians when developed by their own specialty organization [19] and the AAP recently published SSTI treatment recommendations, this is especially concerning. The lack of awareness among our participants of guidelines about a condition that significantly impacts on their practice suggests that more effective methods of dissemination must be identified.

The practice patterns reported by our participants are similar to prior studies of patterns of care for SSTIs showing high rates of antibiotic prescribing, including increased use of antibiotics active against CA-MRSA. In a study using nationally representative office visit data, >75% of children with SSTIs received an antibiotic and use of antibiotics active against CA-MRSA had increased substantially by 2005 [4]. On the other hand, studies examining I&D use have shown low utilization in ambulatory settings [20,21]. A recent survey of pediatric infectious disease specialists reported that 100% recommended antibiotics for SSTIs and many used decolonization regimens, although there was significant variability in antibiotic selection and decolonization methods [22]. Although this variability is an area of concern for our participants, it is notable that the primary prevention strategy recommended by the CDC which emphasizes personal hygiene including hand washing and not sharing personal items was only mentioned by a small number of participants. The CDC recently launched an MRSA Education Campaign (available at ) which will hopefully increase awareness among both health care providers and parents about recognition and prevention of SSTIs caused by CA-MRSA by providing educational materials.

There are several limitations to our study. First, our participants were selected from one urban geographic region and their experiences and opinions may not be generalizable to other communities, including rural areas or those where CA-MRSA is not endemic. Although we used purposive sampling to enhance generalizability and to minimize response bias, it is possible that the study participants' experiences differed from other pediatricians in the region. Although pediatricians provide the majority of pediatric primary care in the United States [23], because we did not include family physicians and emergency department physicians in the study it is unknown whether our findings are applicable to these specialties. Despite these potential limitations, we believe these findings are relevant to primary care physicians nationwide since we included participants from a variety of clinical settings and recent data indicates that SSTIs have increased throughout the country and present most frequently to primary care offices [4].

## Conclusion

Our findings have implications for patient care, healthcare systems and development of SSTI treatment guidelines. Efforts are necessary to improve the dissemination of recommendations focusing on treatment and prevention strategies from the CDC and the AAP to the front-line physicians they are intended to reach. This study suggests also that endorsement by and access to infectious disease specialists and distribution of epidemiologic data focusing on ambulatory patients could promote uptake of new guidelines for SSTIs. Given that most participants were reluctant to not prescribe antibiotics, there is an urgent need to conduct studies rigorously evaluating whether or not antibiotics are necessary for uncomplicated SSTIs and indentifying which patients can be safely treated with I&D alone. Studies evaluating the effectiveness of prevention interventions including decolonization and hygiene are also crucial in order to prevent recurrent and secondary infections which are key drivers of the CA-MRSA epidemic. Additionally, it is necessary to address barriers that interfere with provision of recommended therapies for SSTIs, especially performing I&D. Time constraints and office resources could potentially be addressed by ambulatory procedure clinics. Internal barriers of self-efficacy and knowledge could be addressed through enhanced training opportunities during residency or as continuing education. Our study highlights the complex barriers in improving physician adherence to practice guidelines, especially for practice recommendations targeted at emerging and highly publicized diseases.

## Competing interests

The authors declare that they have no competing interests.

## Authors' contributions

ALH conceived of the study, participated in its design and coordination, analyzed the data and drafted the manuscript. MDC participated in the design and coordination of the study, analysis and interpretation of the data and drafting of the manuscript. RG participated in analysis and interpretation of the data and drafting of the manuscript. BNS participated in the coordination of the study, interpretation of the data and drafting of the manuscript. CSC participated in the design and coordination of the study, analysis and interpretation of the data and drafting of the manuscript. All authors read and approved the final manuscript.

## Pre-publication history

The pre-publication history for this paper can be accessed here:


